# Baculovirus-mediated Gene Delivery and RNAi Applications

**DOI:** 10.3390/v7042099

**Published:** 2015-04-22

**Authors:** Kaisa-Emilia Makkonen, Kari Airenne, Seppo Ylä-Herttulala

**Affiliations:** 1 A.I. Virtanen Institute, Department of Biotechnology and Molecular Medicine, University of Eastern Finland, Kuopio 70211 Finland; E-Mails: Emilia.Makkonen@uef.fi (K.-E.M.); Kari.Airenne@uef.fi (K.A.); 2 Gene Therapy Unit, Kuopio University Hospital, Kuopio 70211, Finland; 3 Science Service Center, Kuopio University Hospital, Kuopio 70211, Finland

**Keywords:** baculovirus, gene transfer, viral vector, RNAi

## Abstract

Baculoviruses are widely encountered in nature and a great deal of data is available about their safety and biology. Recently, these versatile, insect-specific viruses have demonstrated their usefulness in various biotechnological applications including protein production and gene transfer. Multiple *in vitro* and *in vivo* studies exist and support their use as gene delivery vehicles in vertebrate cells. Recently, baculoviruses have also demonstrated high potential in RNAi applications in which several advantages of the virus make it a promising tool for RNA gene transfer with high safety and wide tropism.

## 1. Introduction

Baculoviruses are insect specific viruses that are widely present in nature. They have a long research history [1,2] and a wide variety of data is available about their biology [2] and biosafety [3,4]. The insect specificity of baculoviruses has led in their use as natural insecticides against forestry and agriculture pests [1,5]. The large family of *Baculoviridae* includes over 600 known *lepidoptera*, *hymenoptera* and *diptera* infecting members [6,7,8]. The double-stranded circular supercoiled DNA genome (80–180 kbp) [9,10,11] of the virions is condensed into a nucleoprotein structure known as a core [12]. The core is within a rod-shaped capsid, that is an average of 30–60 nm in diameter and 250–300 nm in length [11,13,14,15] and it is capable of accommodating large DNA inserts [16]. The core and the capsid form the viral nucleocapsid. Membrane-enveloped nucleocapsids form virus particles or virions [17].

Traditionally baculoviruses were divided into two morphologically distinct genera: Nuclear polyhedrosis viruses (NPVs) and granulosis viruses (GVs). New classification divides baculoviruses into four genera: Alphabaculovirus (lepidopteran-specific NPV), betabaculovirus (lepidopteran-specific Granuloviruses), gammabaculovirus (hymenopteran-specific NPV) and deltabaculovirus (dipteran-specific NPV) [6,18]. NPVs are further divided on the basis how they are embedded into viral occlusion bodies (VOBs) comprised of enveloped nucleocapsids in a polyhedrin matrix [19]. They can be packaged as a single nucleocapsid or multiple nucleocapsids per envelope. GVs, on the contrary, have only a single virion within in a very small inclusion body [17].

In nature, baculoviruses have a biphasic infection cycle and two different forms of viruses have specific roles in different stages of the viral life cycle. Occlusion derived virions (ODVs) are responsible for the horizontal transmission of infection between insects by contaminating the soil and plants [20], whereas the budded viruses (BVs) are responsible for the systemic spread of the virus within the insect. The primary infection cycle begins when VOBs enter the insect from a virus-contaminated plant [21,22]. The polyhedrin matrix dissolves in the alkaline environment of the insect midgut [23] and leads to the release of the ODVs. ODVs fuse with the columnar epithelial cells of the digestive tract and enter the midgut cells [24]. While in the cell, the nucleocapsids are transported into the nucleus where the transcription and virus replication take place. During the later lytic cycle of infection, BVs are produced which exit the cell from its basolateral side [25]. These viruses then further spread the infection within the insect through the tracheal system and hemolymph [25,26,27,28,29]. VOBs are produced later on during the infection and the insect dies releasing the VOBs into the environment. ODV and BV do not only have different roles during the infection cycle but they are also structurally different. Though the nucleocapsids of ODV and BV are similar, the virions have different lipid and protein profiles within their envelopes [30]. This is based on the origin of the envelopes. The ODV envelope is derived from the nuclear membrane of the insect cell, whereas the BV envelope is acquired from the host cell membrane [31]. The virions have also different entry mechanisms that they use to enter the host cells. BVs enter cells via endocytosis [32], whereas ODVs enter the midgut epithelial cells by directly fusing with the cell surface membrane [32,33,34,35].

## *2. Autographa californica* Multiple Nucleopolyhedrovirus

*Autographa californica* multiple nucleopolyhedrovirus (AcMNPV), the most extensively studied baculovirus, represents a prototype of the family *Baculoviridae*. The genome of the virus (~134 kbp) has been sequenced and predicted to contain 156 open reading frames, 218 transcription start sites and 120 polyadenylation sites [36,37]. The major capsid protein of the virus is vp39 whereas the major envelope glycoproteins are gp64 (BV) and p74 (ODV). gp64 is involved in the formation of peplomer structures at one end of the rod-shape virion [38]. The basic DNA-binding protein of the virus, p6.9, is responsible for packaging the viral DNA in insect cells [31,39,40]. AcMNPV has been shown to enter insect cells by endocytosis [32,41] in which gp64 has an essential role. The glycoprotein gp64 mediates virus attachment [38] to the cell surface as well as internalization [38] and pH-dependent escape of the virus nucleocapsid from the endosomes [42]. gp64 is also necessary for the production of infective viruses and is important in the virus egress from the cells [43].

The BV of AcMNPV has been broadly used in biotechnology [2,41,44] and the baculovirus expression vector system (BEVS) has become one of the most commonly used methods to produce recombinant proteins [17,45]. Proteins produced with this system include e.g., FDA and EMA accepted vaccine products Cervarix^®^, Provenge^®^ and FluBlok^®^ [46,47,48]. The production of the proteins generally takes place in insect cells derived from *Spodoptera furgiperda* (Sf9 and Sf21AE) and *Trichoplusia ni* (BTI-Tn-5B1-4) [49,50]. These insect cells are able to perform most of the necessary post-translational modifications excluding the N-glycosylation pathway which is more simpler in insect cells [45,51,52]. However recently, an insect cell line SfSWT-5 was developed in which an inducible mammalianized protein N-glycosylation pathway can take place [53]. The BEVS system enables fast and easy production of recombinant proteins, and due to the scalability of the system, the production of large quantities of desired proteins is possible requiring that the genes of interest are placed under the control of strong AcMNPV promoters, such as polyhedrin or p10 promoter [17,54]. In addition to production of various proteins [45], the BEVS has been applied for eukaryotic surface display [55], vaccination [56], drug screening [57], and as well as in production of viral like particles [58] and other gene transfer vectors, such as adeno-associated viruses (AAV) [59] and lentiviruses [60].

## 3. Baculovirus Applications in Vertebrate Cells

Despite being highly insect specific, AcMNPV was well shown to enter human cells already in the 1980s [61]. It however took until 1990s when AcMNPV was shown to efficiently transfer genes into vertebrate cells, especially hepatocytes, if containing an expression cassette including a target cell functional promoter [62,63]. From there on the virus has been successfully utilized in various different types of *in vitro* and *ex vivo* gene transfer applications in many types of dividing and nondividing [15,64,65] vertebrate cell lines, primary cells, progenitor cells, induced pluripontent (iPS) and stem cells [66,67,68]. These comprise a broad spectrum of cells of human, monkey, porcine, bovine, rabbit, rat, mouse, hamster, fish, sheep and avian origin [69,70,71,72,73,74,75,76,77,78] and the list of permissive cells is constantly expanding [69]. Known well-permissive targets for the baculovirus-mediated gene transfer includes cells of the hepatic, kidney and osteosarcoma origin while the poorest are those of hematopoietic origin [70].

The outcome of baculovirus transduction *in vitro* is dependent on several factors and can be enhanced by optimizing transduction conditions and vector design [79,80,81,82,83]. Modifications in the transduction protocol, such as extended incubation time and transduction at temperatures under 37 °C have been shown to lead to enhanced gene expression [61,84,85,86,87,88]. On the other hand, multiple re-additions (supertransduction) of the virus can be used to extend the transient transgene expression [87,89]. Although AcMNPV can enter a variety of cells from different origin, efficient uptake does not necessarily guarantee efficient gene expression [90,91]. One of the important aspects, which has a role in the efficiency of transduction, is the choice of cell culture medium. Commonly used Dulbeccos’s modified Eagle’s medium (DMEM) has been shown to hinder baculovirus-mediated gene delivery [79,80,92] whereas the use of RPMI 1640 medium can yield remarkably better results [80]. Several medium supplements have also been reported to aid in baculovirus-mediated gene delivery and lead to increased gene expression. These include, e.g., histone deacetylase inhibitors like sodium butyrate or trichostatin A [71,93]. Microtubule interfering substances, such as nocodazole or vinblastine, can aid virus-mediated gene delivery as intact microtubule network has been shown to hinder the cytoplasmic transport and nuclear entry of baculoviral nucleocapsid [15,94]. Protein kinase activator Phorbol 12-myristate 13-acetate and DNA methyltransferase inhibitors have also been shown to boost the transgene expression [90,95]. The toxicity of these substances, however, needs to be taken into account in their use [96].

Yet another way to achieve enhanced gene delivery and increase virus tropism is to modify the baculoviral envelope. The major baculoviral envelope glycoprotein, gp64, has been most commonly engineered for this purpose [55,97]. Given the essential role of gp64 in the infection of insect cells and transduction of mammalian cells [98], addition of extra copies of gp64 on the viral envelope has been shown to lead to better transduction efficiency [99]. In addition, gp64 has been engineered to house different peptides or proteins [55,100]. Also, the use of vesicular stomatitis virus envelope G-protein (VSVG) [101] and its truncated version (VSV-GED) has not only led to broadened virus tropism but also to enhanced transduction both *in vitro* as well as *in vivo* [75,99,102,103,104,105]. Additional ways to improve transduction efficiency have included the use of expression targeting ligands [55,106,107,108,109,110,111] and other surface modifications such as avidin [111], biotin [112], lymphatic homing peptide [113], polymer coating with polyethyl glycol [114,115,116,117] and polyethylenimine [118].

In order to achieve most optimal gene expression efficiency, the choice of suitable promoter requires careful selection. CMV and Chicken β-actin promoters are considered as good choices to drive baculovirus-mediated transgene expression in vertebrate cells [73,119]. Baculoviral vectors equipped with cell-type and tissue specific promoters have been successfully used to target transgene expression to certain tissues and cells [120,121,122,123] whereas inducible promoters [123,124] have been used to control the state of the expression. The incorporation of Woodchuck hepatitis virus posttranscriptional regulatory element into the expression cassette has been shown to lead to improved transgene expression in various cell lines [79]. With the aim of prolonging the transient nature of baculovirus-mediated expression, elements enabling long term expression from AAV [125,126], Epstein-Barr virus (EBV) [127] and transposon *Sleeping Beauty* [128] have been incorporated in the virions*.*

Baculoviruses have been shown to promote cytokine production and thus stimulate host antiviral immune responses in mammalian cells [129,130,131,132,133,134,135,136,137]. The virus exposure results in the activation of tumor necrosis factor α, interleukin 1 α, and interleukin 1 β expression along with interferon production [130,131]. The involvement of toll-like receptors has also been suggested since AcMNPV was shown to induce the secretion of tumor necrosis factor α and interleukin 6 along with increased expression of activation ligands in macrophages [129]. The role of toll-like receptor 9 and MyD88-dependent signaling pathway on activation of immune cells via baculoviral DNA has also been demonstrated [132]. However, other viral components and recognition pathways such as toll-like receptor 3 [135] and toll-like receptor-independent routes (interferon regulatory factors 3 and 7) [129,138] seem to be also involved. The induction of antiviral effects in mammalian cells appears to be dependent on cell type [124,136,139] and is transient [140].

## 4. Baculoviral Entry and Trafficking

The efficient entry of AcMNPV into mammalian cells requires multiple successful steps. The process begins with virus binding to the surface of the cells followed by cellular entry, vesicular transport, endosomal escape, cytosolic movement, nuclear entry, capsid disassembly and finally gene expression. The actual entry processes and the exact mechanisms leading to successful infection of insect cells or transduction of mammalian cells are still vaguely understood and somewhat controversial [41,69,70]. Though clathrin-mediated endocytosis is suggested to be involved in the virion uptake into mammalian cells [15,32,141,142,143,144], also the involvement of macropinocytosis, caveolae route or even apoptotic mimicry have been suggested [142,143,144,145]. Among all entry mechanisms, clathrin-independent phagocytosis-like uptake [146] seems to be the most logical since it fits baculovirus biology best. However, the endocytic route used by the virus can also depend on the cell type as well as culture conditions [147].

The wide tropism of the virus suggests that baculovirus utilizes non-specific electrostatic interactions and attaches to the surface of mammalian cells via general cell surface molecule, such as a phospholipid or a heparan sulfate proteoglycan (HSPG) [102,148,149,150]. The involvement of HSPGs is supported by the fact that treatment of HSPGs with heparinase leads to reduction in baculovirus binding [150]. Also, a heparin binding motif was recently detected within gp64 [148]. In addition, it was shown that N- and 6-O-sulfation of HS is vital for baculovirus binding and transduction of mammalian cells. Furthermore Syndecan-1, a ubiquitous proteoglycan, acts as a baculovirus receptor [151]. However, gp64 has also been shown to interact with cell surface phospholipids, such as phosphatidic acid or phosphatidylinositol [102,144,149,152] suggesting that the baculovirus entry and binding are a multistep processes which possibly require several cell surface factors.

The internalization of baculovirus has been shown to be mediated by lipid rafts [144,149] and take place within cholesterol rich areas [144,146,149]. Extensive membrane ruffling has also been associated with virus internalization [146]. The virus uptake has been shown to rely on actin and the trafficking further regulated by Ras homolog gene family member A, ADP-ribosylation factor 6 and dynamin [146]. Differential activation of PKC subtypes α and ε as well as the status of intermediate filament vimentin have all been recently shown to have an effect on baculovirus transduction [80,90]. Following the entry, the virus is transported within vesicles until the pH-dependent fusion of the viral envelope with the endosome releases the capsid to the cytoplasm [41,142,146]. The release is mediated by the major baculoviral type III envelope fusion protein, gp64 [42,153]. The escaped nucleocapsid is then transported in the cell with the aid of actin polymerization and the nucleocapsid enters the nucleus via nuclear pores [154,155,156]. When the virus is in the nucleus, the nucleocapsid disassembles and the viral DNA is released [15,65,91,156]. In the nucleus, baculovirus localizes into discrete foci in the nuclei and induces accumulation of promyelocytic leukemia nuclear bodies [65].

## 5. Animal Studies

Baculovirus offers several advantages as a gene delivery vector compared to other viral vectors in terms of safety, high capacity of carrying foreign DNA, and ease of production. Although the virus does not suffer from pre-existing immunity in vertebrates [157], it is, however, quickly inactivated by serum complement components [62]. This is a result of the classical [158,159] and the alternative [160] pathway activation. This delayed the first successful *in vivo* applications of baculoviruses into late 1990s when the first gene transfer attempt was performed in rats and mice [161]. Today, several different successful gene transfer studies performed in mouse, rat, rabbit and pig animal models have been reported. Logically, the best *in vivo* targets are the immunoprivileged tissues such as the eye [162], the brain [163,164], testis [99,165] or central nervous system [166,167,168]. Approaches to bypass the complement have included the use of complement inactivators, such as soluble complement receptor type 1 [160], cobra venom factor [169] and compstatin [159], which have all proven their usefulness in virus protection. Shielding the viruses with complement interfering factors, such as decay acceleration factor [170], factor H like protein, C4b-binding protein and membrane cofactor have also aided in the battle against the immune system [171].

The first *in vivo* gene transfer attempts with baculovirus were performed in the liver parenchyma of rats and mice but direct systemic and intraportal circulation delivery resulted in undetectable transgene expression [161]. The speculation of the involvement of the immune system in the unsuccessful gene transfer resulted in multiple studies in immune-compromised animals. A direct baculovirus-mediated gene transfer into the liver parenchyma of immunocompromised mice led to detectable transduction of hepatocytes around the injection site [77]. Within the same study, baculovirus injected into nude mice bearing human derived hepatocarcinomas resulted also in low gene transfer efficiency [77]. A systemically performed gene delivery into complement deficient tumor-bearing mice led to transgene expression primarily in liver, spleen, and kidney, but expression was also detected in the tumor [172].

Delivery methods that allow gene transfer in the absence of serum or complement have given better results in immune-competent animals. Direct injection of baculovirus into brain striatum of mice and rats [163,169] led to marker gene expression in the striatum, the corpus callosum, and the ependymal layer. After local delivery of baculovirus into the brain of rats, AcMNPV was found to efficiently transduce cuboid epithelium of the choroid plexus cells. Transgene expression was detected also in endothelial cells of brain microvessels throughout the forebrain [163]. AcMNPV’s tendency to transduce especially well choroid plexus cells has also been verified by other studies performed in rat brain [103,173,174]. Transgene expression could also be detected in the walls of lateral ventricles and in subarachnoid membranes when (VSV-GED) pseudotyped AcMNPV was used [103]. Systemic injection through tail vein with pegylated baculovirus led to enhanced transduction of brain in mice but the transgene expression was also detected in liver, spleen, lung, heart, and kidney [114]. Additionally, a tissue-specific hybrid promoter has been utilized to drive efficient neuron-specific gene expression in rat brain [121,175], as well as glial fibrillary acidic protein promoter for astrocyte-specific gene expression [122]. When transcriptional targeting was used, baculovirus transduction was detectable not only in neurons near the injection sites but also in remote target regions, probably because of axonal transport [176]. Baculovirus containing an expression cassette flanked with the ITRs of AAV extended transgene expression in rat brain [126].

The immunoprivileged nature of the ocular tissue makes it an attractive target for baculovirus-mediated gene therapy. In a direct subretinal administration of baculovirus into mouse eye, a strong expression of the marker gene in retinal pigment epithelial cells followed [177]. Intravitreal injection resulted in marker gene expression in the corneal endothelium, lens, retinal pigment epithelial cells, and retina. When intravitreal gene transfer was performed in rabbits, it resulted in gene expression in the inner retina, photoreceptor cells and in retinal pigment epithelium cells [162]. With the aid of a *Sleeping Beauty* hybrid vector, a long term expression was achieved in mouse eye [178]. In rat retinal vasculature, gene expression has been targeted with human transmembrane *fms*-like tyrosine kinase promoter [179].

Though immunoprivileged areas seem to suit best for baculovirus-mediated gene delivery, direct injection of recombinant baculoviruses into quadriceps femoris muscle of mice resulted in a transient expression of the marker gene [105]. In rats, intramuscularly injected baculovirus was used to treat hyperammonemia [180]. In rabbits, VEGF-D gene transfer was able to efficiently induce angiogenesis in semimembranosus muscle [181]. Baculovirus-mediated enhanced angiogenesis has also been detected in a rat model of ischemic stroke [182] and acute myocardial infarction [183,184], as well as after use of baculovirus based biotherapeutic stent in canine femoral artery [185]. In induced mouse model of liver cirrhosis, intraperitoneal (i.p.) injection of AcMNPV lead to alleviated symptoms via interferon induction [186]. The use of avidin-displaying virus was able to demonstrate extensive expression in rat kidney and spleen after i.p. administration [187]. Hydrodynamic transduction via renal vein resulted in gene expression in kidney [188]. By using a different approach, adventitial cells of carotid arteries of rabbits were successfully transduced by recombinant baculoviruses by using a collar device which allowed minimal exposure to complement [93].

Towards baculoviral applications in cancer gene therapy, an astrocyte-specific baculovirus was successfully used to treat malignant glioma. A virus expressing A-chain of diphtheria toxin effectively suppressed tumor development in a rat xenograft model [189]. When glioblastoma specific promoter (HMGB2) was used to control Herpex simplex thymidine kinase (tk), targeted glioblastoma expression was detected in mouse xenograft model [190]. In the same cancer model, incorporation of miRNA regulation into a GFAP driven *tk* expression improved safety [164]. By combining sodium butyrate with p53 tumor suppressor gene, synergistic results were detected in nude mice bearing human glioma tumors [191]. Several other malignancies which have also been treated with the aid of baculovirus are prostate [128,192,193], ovarian [128], cervical [194], epidermal [117], gastric [195], liver [196,197] and liver metastasis [198], lung [199], melanoma [199,200], and nasopharyngeal cancer [201]. In addition, baculovirus transduced stem cells have functioned *ex vivo* as a targeted delivery vehicles to control tumors [202]. Human embryonic stem cell-derived mesenchymal [203] and neural [204] stem cells as well as mouse [205] and human [206,207] iPS derived neural cells were able to keep cancers in control.

AcMNPV has also demonstrated therapeutic possibilities in other indications besides cancer. For example, efficient transduction of rabbit intervertebral disc has been reported [167]. In another study, lumbar intrathecal injection into the cerebrospinal fluid was used to transduce rat dorsal root ganglia cells [166]. The potential of baculovirus for *ex vivo* cartilage and bone engineering has also been demonstrated in several studies [70]. Transduction of de-differentiated chondrocytes with a baculovirus expressing bone morphogenetic protein-2 (BMB-2) or BMB-2/transforming growth factor β combination was able to restore differentiation status of cells but also increase cartilage-specific extracellular matrix formation [208,209,210]. The cells were able to grow into cartilage when seeded in polymeric scaffold in a bioreactor [211]. In addition, osteochondral defects have been healed with produced cartilage implants in rabbits [212]. BMB-2 transduced and implanted BMSCs were able to induce bone formation in mice and promote bone repair in rats [213]. Implantation of BMB-2 and VEGF expressing BMSC cells into segmental bone defects in rabbits led to accelerated bone healing [214]. Adipocyte stem cells (ASCs) can also serve as promising cells in bone regeneration [215,216,217,218]. The safety of baculovirus in tissue engineering has been supported by several studies which have shown that baculovirus neither altered *HLA-II* expression nor impaired the immunosuppressive nature of BMSCs and induced only mild and transient immune response without disrupting the karyotype [219]. When Hybrid BV-AAV vectors were used to transduce rat-derived BM-MSCs, high transgene expression was achieved and no cytotoxicity was reported [220].

The unfortunate side effects of gene transfer include the risk of insertional mutagenesis and cancer with retroviruses [221], and humoral and cellular immune responses in the case of adenoviruses [222]. Compared to these viral vectors, baculoviruses are safer since they are unable to replicate and cause diseases outside non-vertebrate hosts [4], there is no pre-existing immunity in vertebrates [157] and the viruses are unable to integrate into host cell genome. In addition, the viruses have the capacity to transfer large genes [17] and the scalability of the production makes them an attractive tool for gene transfer. Although baculoviruses have been extensively studied throughout the years, no clinical data is yet available of their therapeutic use. However, the BV system has already been approved to be clinically suitable for vaccine and AAV vector production (Glybera) purposes by FDA and EMA and encouraging results in cancer treatment are expected to lead to the first clinical trial in the near future [223].

## 6. Baculovirus and RNAi

Though RNA interference (RNAi) is a relatively new discovery, it has already become a potent and specific method for gene regulation. Gene silencing by RNA interference can be used when loss-of-function studies with sequence specific knock-down of gene expression are needed in different biological situations [224,225]. It also enables a new and promising approach to treat common diseases and thus provides a convenient tool for analysis of gene function, as well as gene therapy [226,227]. Baculoviruses are highly viable alternatives for RNAi delivery since they are very inert and versatile [228,229] with possibilities of high throughput preparation [173,230].

RNAi has been shown to have an important regulatory role in insects [231] in which various gene silencing studies have already been carried out [232,233]. Thus far, four *Bombyx mori* nucleopolyhedrosis virus (BmNPV)-encoded miRNAs have been identified which are evolutionarily conserved among many baculoviruses [234]. In *S. frugiperda*, differential expression of several miRNAs upon baculovirus infection has been detected [235]. The miRNA profile of *Helicoverpa armigera* larvae has also been shown to alter upon *H. armigera* single nucleopolyhedrovirus (HaSNPV) infection [236]. The RNAi-approach has been successfully used to prevent AcMNPV infection *in vitro* and *in vivo* [237] as well as in the prevention of BmNPV infection in *B. mori* cells and in the silkworm *B. mori* [238,239,240]. BmNPV has been shown to encode miRNAs which modulate the small-RNA-mediated defense as well as regulate the expression of DNA binding protein (P6.9) and other late genes vital for the late stage of viral infection in its host *Bombyx mori* [241,242].

AcMNPV encodes miRNAs that lead to a reduction of BVs and accelerated formation of ODVs [243]. The RNAi has been shown to persists for four to eight days in baculovirus-infected as well as uninfected Sf9 cells [232].The RNAi-approach has been also used to increase recombinant protein production in a *Trichoplusia ni* derived cell line (BTI-TN-5B1-4-High Five) [244].

The first RNAi application in mammalian cells was demonstrated in a study where baculovirus-delivered U6-driven short hairpin RNA (shRNA) designed against lamin A/C led to effective knockdown of the corresponding mRNA and protein levels in Saos2, HepG2, Huh7, and primary human hepatic stellate cells [245]. In another study, a shRNA under the control of hybrid CMV enhancer-H1 promoter was capable of suppressing the expression of the target luciferase gene by 95% in cultured rat glioma C6 cells, up to 80% in human NT2 neural precursor cells and 82% in rat brain *in vivo* [246]. In addition, baculovirus delivered miRNA has been shown to repress efficiently the overexpression of endogenous TNF-α in arthritic synoviocytes. In the same study, a hybrid baculovirus vector containing miRNA combined with *Sleeping Beauty* transposon was shown to effectively repress the transgene expression for prolonged periods in HEK293 cells [247]. *Sleeping Beauty* transposon containing baculovirus was also recently coupled with PTENP1 long non-coding RNA (lncRNA) which inhibited cell proliferation in hepatocellular carcinoma cells (HCC) *in vitro* and HCC tumors in mice [248]. *Sleeping Beauty* hybrid vector was also used to deliver miRNAs 122 and 155 into HCC cells *in vitro* and into HCC tumor *in vivo* with the result of HCC growth inhibition [197]. Silencing of miRNA-10b by baculoviral decoy vectors *in vitro* in U87-M21 glioma cell line led to reduced growth, invasion and angiogenesis of the cells. *In vivo*, the inhibition of miRNA-10b in human glioma mouse model diminished the invasiveness, angiogenicity, and growth of the tumor [249]. Recently, baculovirus was harnessed for miRNA-26a, -29b, -148b and -196a delivery and resulted in improved hASCs osteogenesis [250].

Several baculoviral vectors have been engineered to inhibit the replication of multiple pathogenic viruses. These include a baculovirus expressing shRNAs against *peste des petits ruminants* virus. Within the study, a successful inhibition of the generation of infectious progeny was observed *in vitro* in Vero cells [251]. A VSVG pseudotyped baculovirus containing U6 promoter driven shRNA targeting arterivirus porcine reproductive and respiratory syndrome virus (PRRSV) genome resulted in the inhibition of viral replication in Marc145 cells [252]. In addition, a baculovirus based shRNA against the highly conserved core region of the hepatitis C virus (HCV) genome was able to inhibit the expression of the HCV core protein and thus virus replication in NNC#2 cells [253]. Long-term expression of shRNA against the highly conserved core-protein region of HCV was achieved with hybrid baculovirus containing EBV EBNA1 and OriP sequences. Inhibition of HCV core protein lasted for at least 14 days [254]. When HepG2 cells were transduced with baculovirus bearing shRNA against hepatitis B virus (HBV), a reduction in the formation of HBV covalently closed circular DNA was detected [255]. Baculovirus-delivered bispecific shRNA has also markedly inhibited the production of influenza viruses A and B [256]. Interestingly, a VSVG pseudotyped baculovirus vector carrying a ribozyme-synthesizing cassette under the tRNA(i)(Met) promoter was constructed. Transduction of HeLa CD4(+) cells with the HIV-1 U5 gene-specific ribozyme suppressed HIV-1 expression within the cells [257].

In conclusion, this review summarizes the wide applications of baculovirus (AcMNPV) in gene transfer. The current knowledge of the efficacy and safety along with numerous advantages as gene delivery vehicles support AcMNPV use also for RNAi applications Table 1).

**Table 1:** Summary of baculovirus mediated preclinical RNAi studies in vertebrate cells.

Cell/Tissue	Promoter	RNAi/Target	*In Vitro/In Vivo*	Ref., Year
Saos2, HepG2, Huh7, primary hepatic stellate cells	U6	shRNA; Lamin A/C	*In vitro*	[245], 2005
C6, NT2, rat brain	CMV enhancer/H1 promoter	shRNA; Luciferase	*In vitro*, *in vivo*	[246], 2005
Marc145	U6	shRNA; PRRSV nucleoprotein	*In vitro*	[252], 2006
HeLa CD4+	tRNA_i_^Met^	Ribozyme; U5 region of HIV LTR	*In vitro*	[257], 2006
NNC#2	U6	shRNA; HCV core protein	*In vitro*	[253], 2008
NNC#2	U6	shRNA; HCV core protein. EBNA1 and OriP for prolonged expression.	*In vitro*	[254], 2009
MDCK	U6	shRNA; influenza nucleoproteins	*In vitro*	[256], 2009
HepG2	U6	shRNA; HBV genome	*In vitro*	[255], 2009
HEK293, synoviocytes	CMV	miRNA; EGFP, TNF-α. *Sleeping Beauty* for prolonged expression.	*In vitro*	[247], 2011
Vero	U6	shRNA; nucleoprotein of PPRV.	*In vitro*	[251], 2011
U87-M21, U87-M21 tumor in mice	CMV	miRNA-10b for inhibition of growth, invasion and angiogenesis.	*In vitro*, *in vivo*	[249], 2012
ASC, calvarial bone defects in mice	CMV	miRNA-26a, -29b, -148b, -196a for promoting osteogenic differentiation.	*In vitro*, *in vivo*	[250], 2014
HCC Mahlavu, HCC tumor in mice	CMV	lncRNA; PTENP1. *Sleeping Beauty* for prolonged expression.	*In vitro*, *in vivo*	[248], 2015
HCC Mahlavu, HCC tumor in mice	CMV	miRNA-122, -151 to combat HCC tumorigenity/metastasis. *Sleeping Beauty* for prolonged expression.	*In vitro*, *in vivo*	[197], 2015
